# Willin/FRMD6 Influences Mechanical Phenotype and Neuronal Differentiation in Mammalian Cells by Regulating ERK1/2 Activity

**DOI:** 10.3389/fncel.2020.552213

**Published:** 2020-09-04

**Authors:** Nils M. Kronenberg, Andrew Tilston-Lunel, Frances E. Thompson, Doris Chen, Wanjia Yu, Kishan Dholakia, Malte C. Gather, Frank J. Gunn-Moore

**Affiliations:** ^1^Centre of Biophotonics and SUPA, School of Physics and Astronomy, University of St Andrews, St Andrews, United Kingdom; ^2^Centre for Nanobiophotonics, Department of Chemistry, University of Cologne, Cologne, Germany; ^3^Centre of Biophotonics, School of Biology, University of St Andrews, St Andrews, United Kingdom; ^4^Department of Biochemistry, School of Medicine, Boston University, Boston, MA, United States; ^5^Department of Physics, College of Science, Yonsei University, Seoul, South Korea

**Keywords:** Willin/FRMD6, neuronal differentiation, ERK1/2, cell mechanics, actin, focal adhesion, TAZ, cell force measurement

## Abstract

Willin/FRMD6 is part of a family of proteins with a 4.1 ezrin-radixin-moesin (FERM) domain. It has been identified as an upstream activator of the Hippo pathway and, when aberrant in its expression, is associated with human diseases and disorders. Even though Willin/FRMD6 was originally discovered in the rat sciatic nerve, most studies have focused on its functional roles in cells outside of the nervous system, where Willin/FRMD6 is involved in the formation of apical junctional cell-cell complexes and in regulating cell migration. Here, we investigate the biochemical and biophysical role of Willin/FRMD6 in neuronal cells, employing the commonly used SH-SY5Y neuronal model cell system and combining biochemical measurements with Elastic Resonator Interference Stress Micropscopy (ERISM). We present the first direct evidence that Willin/FRMD6 expression influences both the cell mechanical phenotype and neuronal differentiation. By investigating cells with increased and decreased Willin/FRMD6 expression levels, we show that Willin/FRMD6 not only affects proliferation and migration capacity of cells but also leads to changes in cell morphology and an enhanced formation of neurite-like membrane extensions. These changes were accompanied by alterations of biophysical parameters such as cell force, the organization of actin stress fibers and the formation of focal adhesions. At the biochemical level, changes in Willin/FRMD6 expression inversely affected the activity of the extracellular signal-regulated kinases (ERK) pathway and downstream transcriptional factor NeuroD1, which seems to prime SH-SY5Y cells for retinoic acid (RA)-induced neuronal differentiation.

## Introduction

Willin/FRMD6 is part of a family of proteins in which a 4.1 ezrin-radixin-moesin (FERM) domain has been identified (Gunn-Moore et al., 2005). Members of the FERM protein family have been linked to a variety of signaling pathways and their aberrant expression is associated with human diseases and disorders (Moleirinho et al., 2013a; Gunn-Moore et al., 2016). Specifically, Willin/FRMD6 is an upstream component of the Hippo signaling pathway that can regulate the activity of the transcriptional factor YAP/TAZ to influence cell morphology and behavior (Angus et al., 2012; Moleirinho et al., 2013b).

Willin/FRMD6 has been indirectly linked to the nervous system, for example, it was first isolated from the rat sciatic nerve (Gunn-Moore et al., 2005), where it controls the activity of fibroblasts (Moleirinho et al., 2013c) and potentially influences myelination (Fernando et al., 2016). Also, single nucleotide polymorphisms associated with the frmd6 gene have been associated with hippocampal atrophy and Alzheimer’s disease in the Alzheimer’s Disease Neuroimaging Initiative (ADNI; Hong et al., 2012; Shen et al., 2014; Nho et al., 2016). Despite these findings, most studies to date have focused on Willin/FRMD6 functional roles in cells outside of the nervous system, where in addition to influencing the Hippo pathway, Willin/FRMD6 is involved in forming apical junctional cell-cell complexes through interaction with Par3, atypical protein kinase C (aPKC) and Par6 (Ishiuchi and Takeichi, 2011). As well as these links between Willin/FRMD6 and the cellular architecture, it is now becoming apparent that other components of the Hippo signaling pathway are linked to not only transducing but also changing the mechanical properties of cells and so can influence their surrounding physical environment (Totaro et al., 2018).

In this report, we sought to shed light on the biochemical and biophysical role of Willin/FRMD6 in the commonly used SH-SY5Y neuronal model cell system (Kovalevich and Langford, 2013). SH-SY5Y cells are a widely used neuroblastoma cell line that is proliferative and can differentiate into neuronal-like cells using chemical agents such as retinoic acid (RA; Encinas et al., 2000). They exhibit Willin/FRMD6 expression levels identical to primary mouse cortical neurons (Supplementary Figure S1) and are thus well suited as a model for investigation of Willin/FRMD6 in a neuronal setting. As well as looking at the effect of Willin/FRMD6 on key components of these cells, we used Elastic Resonator Interference Stress Microscopy (ERISM; Kronenberg et al., 2017), to study the involvement of Willin/FRMD6 in biophysical aspects of SH-SY5Y cells. ERISM is a recently developed force mapping tool that allows for imaging of cell forces with high spatial resolution.

Combining ERISM with biochemical measurements, we discovered that manipulating Willin/FRMD6 expression levels in SH-SY5Y cells influences both biochemical and biophysical effects. Reducing endogenous Willin/FRMD6 expression resulted in a change in proliferation rate and cell morphology, specifically an increase in fine membrane extensions, which in turn rendered the cells more susceptible to differentiation following RA stimulation. These cellular changes were accompanied by changes in the forces that the cells exerted onto their environment. Furthermore, changes in Willin/FRMD6 expression resulted in alterations of the actin cytoskeleton and the localization of TAZ; though notably these events were controlled by activation of the extracellular signal-regulated kinases (ERK) pathway. These combined biochemical and physical measurements provide the first direct evidence that Willin/FRMD6 has potential roles in the development of neuronal-like cells and that these occur predominantly through the ERK signal transduction pathway.

## Materials and Methods

### Cell Culture

SH-SY5Y cells were obtained from ATCC (CRL-2266) and cultured according to ATCC guidelines. The SH-SY5Y cell line consists of a heterogeneous population that is predominantly composed of N-type cells with a small fraction of S-type cells. According to previous reports, N-type cells have faster proliferation rates when compared to S-type cells (Bell et al., 2013). All experiments in this study were performed on mixed cultures within 20 cell doublings. For the generation of the SH-SY5Y cells used in knockdown studies, lentiviral particles containing shRNAs designed to silence Willin mRNA (*shWillin*: 19mer target sequences: 5’-ACAGAGCAGCAAGATACTA-3’) or a scrambled RNA sequence (*shScr*) were produced in HEK293T cells as previously described (Angus et al., 2012). To generate SH-SY5Y cells overexpressing Willin (*Willin*) and reference cells (*Vector*), retroviral particles were generated in Phoenix A cells as previously described (Tilston-Lünel et al., 2016). Antibiotic selection was maintained for 3 weeks of post-viral transduction.

### Western Blot Analysis

Lysates of cell monolayers were prepared using lysis buffer (50 mM Tris-HCl, pH 8.0, 150 mM NaCl, 1.0% Triton X-100, 0.5% sodium deoxycholate, 0.1% sodium dodecyl sulfate (SDS); all reagents purchased from Sigma–Aldrich), and lysate protein concentrations measured by BCA assay. Protein samples prepared with 20 μg of protein lysate and 5× loading buffer (500 mM dithiothreitol, 250 mM Tris-HCl, pH 6.8, 50% glycerol, 10% SDS, 0.25% bromophenol blue) were denatured by heating at 95°C for 5 min and separated by SDS-PAGE. Proteins were then transferred in a liquid buffer (25 mM Tris base, pH 8.3, 192 mM glycine, 20% (v/v) methanol) onto a PVDF membrane (EMD Millipore) using a Bio-Rad blotting module at 25 V for 90–120 min. Membranes were stained with Ponceau S solution (Sigma–Aldrich) to verify effective protein transfer, then washed with TBS-T (20 mM Tris base, pH 7.6, 150 mM NaCl, 0.1% Tween-20). Membranes were blocked in either 5% skimmed dried milk in TBS-T or 5% BSA in TBS-T for 1 h at room temperature, before being incubated with primary antibody diluted in blocking solution overnight at 4°C [FRMD6: Cell Signaling Technology, 14688, 1:100; Phospho-p44/42 MAPK: Cell Signaling Technology, 4370, 1:4,000; p44/42 MAPK (ERK1/2): Cell Signaling Technology, 9107, 1:2,000; NeuroD1: Santa Cruz Biotechnology, sc-1084, 1:100; Vinculin: Novus Biologicals, NBP2-41274, 1:500; β-actin: Sigma–Aldrich, A1978, 1:20,000; GAPDH: Sigma–Aldrich, G8795, 1:20,000]. Membranes were then washed three times with TBS-T for 10 min and incubated with horseradish peroxidase (HRP)-conjugated secondary antibody diluted in blocking solution for 1 h at room-temperature (Abcam, ab6789, 1:10,000; Abcam, ab97051, 1:10,000; LI-COR, 925-32210, 1:25,000; LI-COR, 925-68071, 1:25,000). Membranes were washed with TBS-T for 15 min three times, and protein bands were detected by the addition of 0.75 ml Immobilon Western Chemiluminescent HRP Substrate for 1 min. Protein bands were visualized with the Fujifilm LAS-3000 imaging system and analyzed using FIJI. Alternatively, membranes were incubated with fluorescently labeled secondary antibodies (Li-Cor) and bands detected using the Li-Cor Odyssey CLx (Li-Cor). Bands of the protein of interest were quantified using GAPDH or β-actin as a loading control.

### qPCR Analysis

Quantitative PCR of the synthesized cDNA was conducted using Brilliant II SYBR^®^ Green QPCR Master Mix (Agilent) or Brilliant III Ultra-Fast SYBR^®^ Green QPCR Master Mix (Agilent) according to the manufacturer’s protocol. qPCR reactions were performed on a Roto-Gen Q Series (activation of the polymerase at 95°C for 600 s; denaturation at 95°C for 30 s, 40 cycles; annealing and elongation at 60°C for 60 s; melt curves: 55–95°C at 30 s per 0.5°C) and analyzed using Rotor-Gene Q Series Software. No template controls reactions using nuclease-free water were run for every primer pair during every run to evaluate the background signal. Relative changes in gene expression were calculated according to the 2^−ΔΔCT^ method. The sequences of the qPCR primers are described in Supplementary Table S1.

### Cell Migration Assay Using a Boyden Chamber

A serum-free SH-SY5Y cell suspension (5 × 10^5^ cells) was added to cell culture inserts with 8.0 μm pores (BD Biosciences, UK). Growth media supplemented with 20% serum was added to the lower chamber. The bottom side was stained with 0.3% crystal violet (Sigma–Aldrich). The number of cells on the lower surface of each chamber was counted from images acquired using a Zeiss Axiovert 40CFL microscope.

### ERISM Measurements

ERISM substrates with an apparent stiffness of 3 kPa were fabricated as described previously (Kronenberg et al., 2017) and four silicon chambers (surface area: 0.75 × 0.75 cm^2^; Ibid) were applied. The substrate was incubated with a type I collagen suspension (Collagen A, Biochrome) at pH 3.0–3.5 for 1 h at 37°C and then washed with cell culture medium. The different SH-SY5Y cell lines (*shScr*, *shWillin*, *Vector*, and *Willin*) were investigated in different wells on the same ERISM chip. Cells were seeded onto the ERISM substrate at 3,000 cells per well and kept at 37°C, 5% CO_2_ culture conditions in complete growth media for 24 h to allow adhesion to complete. ERISM measurements were performed and displacement maps were computed as previously described in Kronenberg et al. (2017). For the Fourier-filtered ERISM map, an FFT band-pass filter with an upper cut-off frequency of 3.2 μm was applied to the raw displacement maps using FIJI. For cell force analysis, “indented volume” was calculated by numerically integrating the displacement below −10 nm under each cell (Kronenberg et al., 2017).

### Cell Morphology

The area of cells was measured from phase-contrast images taken 24 h after seeding the cells on ERISM substrates. For this, the outlines of the cells were drawn by hand using the FIJI software package and the area of the marked region was calculated. Cell elongation factors were calculated from the same images by measuring the longest (*l*) and shortest (*s*) diameter of a cell and calculating the elongation as (1 – *s*/*l*).

Cell membrane extension lengths were determined by hand using the FIJI software package. All cells possessing at least one extension with a length at least twice the cell body diameter were classified as a neurite-like extension-bearing cell (“pre-neurite”; Popovics et al., 2013). Results were expressed as the percentage of fine extension bearing cells in the entire cell ensemble.

### Immunostaining

For immunochemistry staining, cells were either fixed directly on ERISM substrates or coverslips. Fixation was achieved with 4% paraformaldehyde (Alfa Aesar) in PBS at room temperature for 20 min. Cells were washed twice with 0.05% Tween-20 in PBS (Alfa Aesar), permeabilized with 0.1% Triton X-100 (Alfa Aesar) in PBS for 3 min, washed twice again, and blocked for 30 min with 1% BSA (Carl Roth) in PBS. Fixed cells on ERISM chips were simultaneously incubated with primary antibodies for vinculin (Merck Millipore, 90227, 1:250) and TAZ (Sigma–Aldrich, HPA007415, 1:100) in blocking solution for 60 min at RT, washed three times, incubated with the secondary antibodies (Sigma–Aldrich, F0257, 1:32; Jackson ImmunoResearch, 711-175-152, 1:400) and TRITC-conjugated phalloidin (Merck Millipore, 90228, 1:500) in blocking solution for 45 min at RT, washed three times, and stained with DAPI (Merck Millipore, 90229, 1:1,000 in blocking solution for 3 min at RT). Fixed cells on coverslips were incubated with a primary antibody for neuroD1 (Santa Cruz Biotechnology, sc-1084, 1:50 in blocking solution, overnight at 4°C), washed, incubated with a secondary antibody (Jackson ImmunoResearch, 205-482-176, 1:1,000 in PBS for 30 min at RT) and washed again. *Antifade Mountant* with DAPI (Invitrogen; P36935) was used to visualize cell nuclei.

### Differentiation of SH-SY5Y Cells

SH-SY5Y cells were plated on the ERISM substrate or glass coverslip and incubated for 24 h. Cells then underwent two gentle washes of PBS to remove any excess serum leftover from the growth media before the addition of SH-SY5Y differentiation media [DMEM:F12, 1% FBS, 1% Penicillin/Streptomycin, 10 μM (RA, 10 mM in EtOH)]. Fresh differentiation media was added to the cells every 2 days for a period of 7 or 8 days. Differentiated cells were defined as cells with neurites that were longer than 40 μm.

## Results

### Knock-Down of Willin/FRMD6 Affects Proliferation, Migration, Morphology and Force Exertion of SH-SY5Y Cells

**Figure 1:**
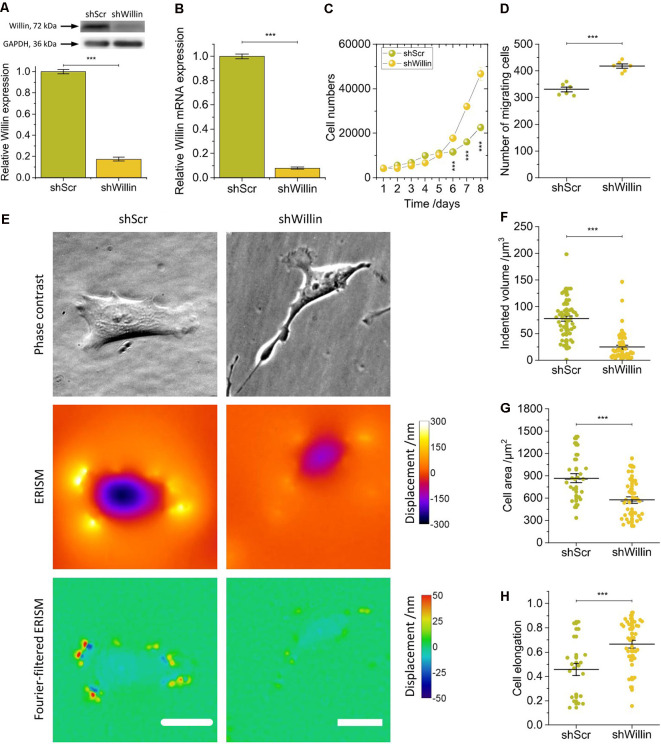
Knock-down of Willin/FRMD6 affects proliferation, migration, morphology, and force exertion of SH-SY5Y cells. **(A)** Quantitative Western blot analysis of Willin/FRMD6 expression in control (*shScr*) and Willin/FRMD6 knock-down (*shWillin*) SH-SY5Y cells. Means were calculated from three independent experiments. Error bars represent SEM. GAPDH serves as a loading control of the lysates. **(B)** qPCR analysis of Willin/FRMD6 mRNA expression in *shScr* and *shWillin* cells. Means and SEM (error bars) were calculated from two independent experiments, each of which was conducted in triplicates.** (C)** Growth curve of *shScr* and *shWillin* cells. Means (horizontal lines) and SEM (error bars) were calculated from three independent experiments, each of which was conducted in triplicates.** (D)** Assessment of migration of *shScr* and *shWillin* cells in Boyden chambers after 24 h. Means (horizontal lines) and SEM (error bars) were calculated from two independent experiments, each of which was conducted in triplicates.** (E)** Phase-contrast images (upper row), ERISM displacement maps (middle row), and Fourier-filtered ERISM maps (lower row) of representative *shScr* (left column) and *shWillin* (right column) cell. Scale bars: 25 μm. Comparison of **(F)** volume by which cells indent into the ERISM substrate, **(G)** cell area, and **(H)** cell elongation of *shScr* and *shWillin* cells. Each data point represents the measured value for one cell taken from four **(F)** and two **(G,H)** independent experiments, respectively, lines indicate means, error bars SEM. Groups were compared using Student’s *t*-test; ***p ≤ 0.001.

We generated an SH-SY5Y cell line (*shWillin*) with a reduced expression level of endogenous Willin/FRMD6 using a short hairpin interference construct and a control cell line (*shScr*) with a scrambled short hairpin interference construct. Successful generation was confirmed by Western blotting (mean relative Willin/FRMD6 expression ± SEM: *shScr*: 1.00 ± 0.02, *n* = 3; *shWillin*: 0.17 ± 0.02, *n* = 3; Student’s* t*-test: *p* ≤ 0.001; Figure 1A) and qPCR analysis (mean relative Willin/FRMD6 mRNA expression ± SEM: *shScr*: 1.00 ± 0.02, *n* = 6; *shWillin*: 0.08 ± 0.01, *n* = 6; Student’s *t*-test: *p* ≤ 0.001; Figure 1B).

A decrease in Willin/FRMD6 expression increased proliferation (mean cell number after 8 days ± SEM: *shScr*: 22,600 ± 600, *n* = 9; *shWillin*: 46,700 ± 1,700, *n* = 9; Student’s *t*-test: *p* ≤ 0.001) and migration capacity (mean number of migrating cells ± SEM: *shScr*: 331 ± 9, *n* = 6; *shWillin*: 417 ± 8, *n* = 6; Student’s *t*-test: *p* ≤ 0.001; Figures 1C,D) of SH-SY5Y cells.

To investigate if Willin/FRMD6 knockdown also led to changes in cellular force exertion, *shScr* and *shWillin* cells were seeded on ERISM substrates and investigated after letting them firmly adhere to the substrate for 24 h. Figure 1E shows phase contrast images of representative *shScr* and *shWillin* cells as well as ERISM maps which show the deformation of the mechanical activity of the cells caused to their soft substrate. Taking the volume by which the cells indent into the ERISM substrate as a proxy for the magnitude of the exerted force, Willin/FRMD6 knockdown resulted in a significant reduction of cell force [mean indented volume ± SEM: *shScr*: (77 ± 5) μm^3^, *n* = 50; *shWillin*: (25 ± 3) μm^3^, *n* = 57; Student’s *t*-test: *p* ≤ 0.001; Figures 1E,F]. Spatial Fourier-filtering was used to filter out broad deformation features from the ERISM displacement maps to resolve finer details of the mechanical interactions of the cells with the substrate. The Fourier-filtered ERISM displacement maps in Figure 1E show substrate twisting at the periphery of both cells. We have previously shown that twisting of ERISM substrates is caused by contractile actomyosin forces that are transmitted to the substrate by focal adhesion complexes (Kronenberg et al., 2017). The number of focal adhesion contacts and the magnitude of substrate twisting, and thus exerted force, was reduced for *shWillin* cells, again indicating a reduction in cell force upon Willin/FRMD6 knockdown.

As evident from Figure 1E and Supplementary Figure S2, reduction in expression of Willin/FRMD6 also led to morphological changes in *shWillin* cells, which manifested as an increase in the number of small fine extensions (see discussion of Figure 4 below), a decrease in cell area [mean cell area ± SEM: *shScr*: (867 ± 63) μm^2^, *n* = 24; *shWillin*: (573 ± 41) μm^2^, *n* = 37; Student’s *t*-test: *p* ≤ 0.001; Figure 1G] and an increase in cell elongation (mean cell elongation ± SEM: *shScr*: 0.46 ± 0.05, *n* = 20; *shWillin*: 0.66 ± 0.03, *n* = 37; Student’s *t*-test: *p* ≤ 0.001; Figure 1H).

**Figure 2:**
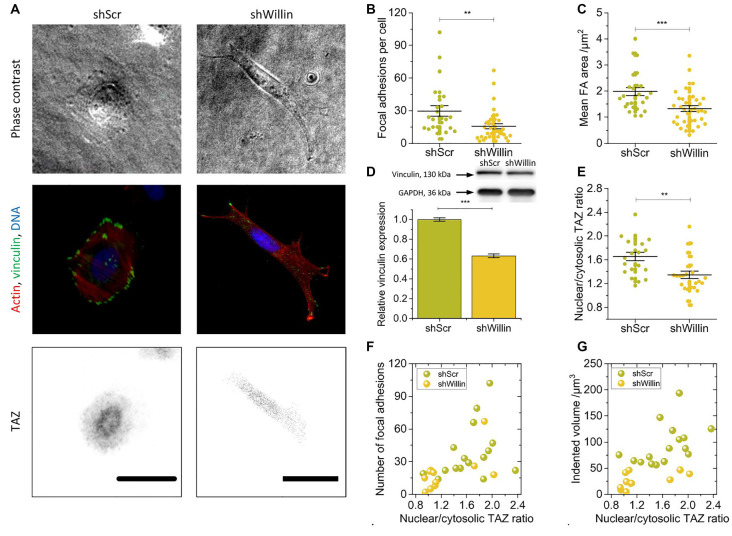
Knock-down of Willin/FRMD6 alters vinculin expression, focal adhesion assembly, and actin cytoskeleton dynamics.** (A)** Representative phase-contrast images (upper row), immunostainings against F-actin (red), vinculin (green) and DNA (blue; middle row) and TAZ (lower row) of control (*shScr*) and Willin/FRMD6 knock-down (*shWillin*) SH-SY5Y cells. Scale bars: 30 μm. Comparison of **(B)** the number of focal adhesions per cell, **(C)** the mean area of a single focal adhesion, and **(E)** the nuclear/cytosolic TAZ ratio in *shScr* and *shWillin* cells. Each data point in **(B)**, **(C)** and **(E)** represents the measurement value for one cell taken from two independent experiments. Lines indicate means, error bars SEM. **(D)** Quantitative Western blot analysis of vinculin expression in *shScr* and *shWillin* cells. Means were calculated from three independent experiments. Error bars represent SEM. GAPDH serves as a loading control of the lysates. Correlation between the ratio of nuclear to cytoplasmic TAZ concentration, **(F)** the number of focal adhesions, and **(G)** the volume by which cells indent into the ERISM substrate for *shScr* (green dots) and *shWillin* (yellow dots) SH-SY5Y cells. Groups were compared using Student’s *t*-test; **p ≤ 0.01; ***p ≤ 0.001.

**Figure 3:**
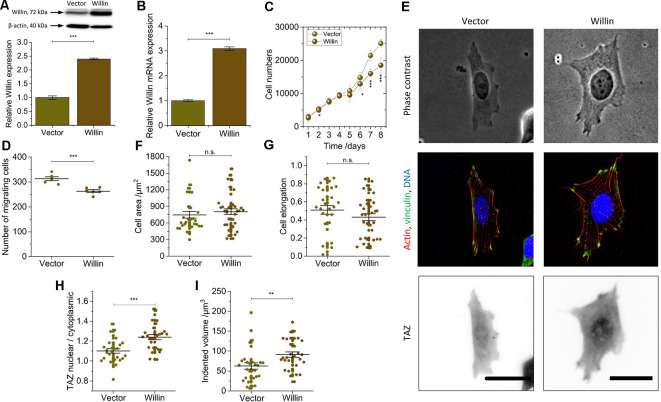
Willin/FRMD6 over-expression results in a general reversal of the findings observed for knock-down cells and activate the mechanical activity of SH-SY5Y cells.** (A)** Quantitative Western blot analysis of Willin/FRMD6 for control (*Vector*) and Willin/FRMD6 overexpressing (*Willin*) SH-SY5Y cells. Means and SEM (error bars) were calculated from two independent experiments, each of which was conducted in triplicates. β-actin serves as a loading control of the lysates. **(B)** qPCR analysis of Willin/FRMD6 expression in *Vector* and *Willin* cells. Means and SEM (error bars) were calculated from two independent experiments, each of which was conducted in triplicates. **(C)** Growth curve of *Vector* and *Willin* cells. Means and SEM (error bars) were calculated from three independent experiments, each of which was conducted in triplicates. **(D)** Assessment of migration of *shScr* and *shWillin* cells in Boyden chambers after 24 h. Means (horizontal lines) and SEM (error bars) were calculated from two independent experiments, each of which was conducted in triplicates.** (E)** Representative phase-contrast images (upper row), immunostainings against F-actin (red), vinculin (green) and DNA (blue; middle row), and TAZ (lower row) of *Vector* and *Willin* SH-SY5Y cells. Scale bars: 30 μm. Comparison of **(F)** cell area, **(G)** cell elongation, **(H)** nuclear/cytosolic TAZ ratio, and **(I)** volume by which cells indent into the ERISM substrate for *Vector* and *Willin* cells. Each data point represents the value measured for one cell. Lines indicate means, error bars SEM. Groups were compared using Student’s *t*-test; ns: *p* > 0.05; *p ≤ 0.05; **p ≤ 0.01; ***p ≤ 0.001.

**Figure 4:**
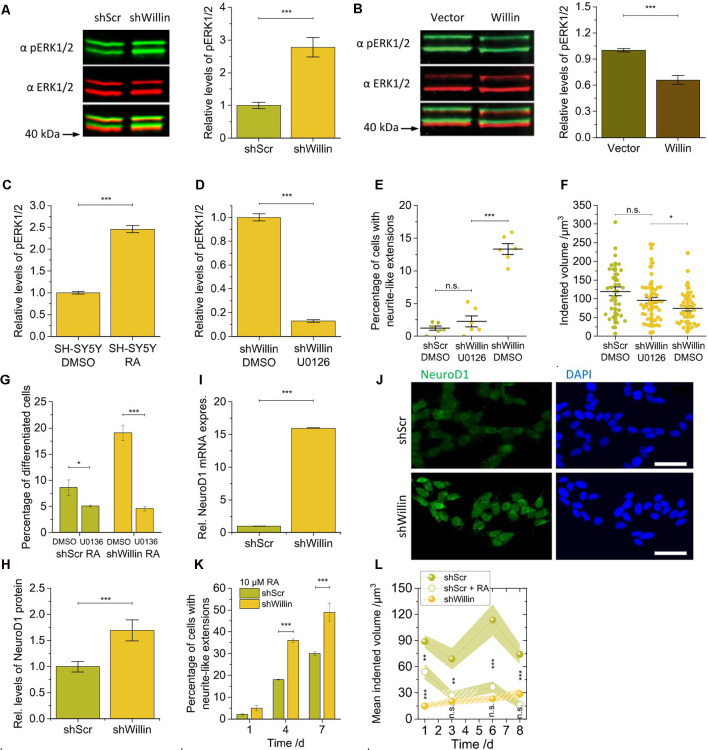
Knockdown of Willin/FRMD6 in SH-SY5Y cells activates the ERK1/2 pathway and elicits differentiation into a neuronal phenotype by activation of the transcriptional factor NeuroD1. Quantitative Western blot analysis of the levels of pERK1/2 relative to ERK1/2 **(A)** in control (*shScr*) and Willin/FRMD6 knock-down (*shWillin*) SH-SY5Y cells, **(B)** in control (*Vector*) and Willin/FRMD6 overexpressing (*Willin*) SH-SY5Y cells, **(C)** in SH-SY5Y wild type cells treated with either DMSO or retinoic acid (RA) and **(D)** in SH-SY5Y wild type cells treated with either DMSO or U0126. Means in **(A)** to **(D)** were calculated from three technical repeats. Error bars represent SEM. Comparison of **(E)** percentage of fine extension-bearing cells and **(F)** volume by which cells deform the ERISM substrate (each data point represents the value measured for one cell) for *shScr* cells treated with DMSO and *shWillin* cells treated with either DMSO or U0126 for 24 h. In **(E)** means were calculated from two independent experiments conducted in triplicate. In **(F)** each data point represents the value measured for one cell. Lines indicate means, error bars SEM.** (G)** Percentage of differentiating *shScr* and *shWillin* cells quantified from bright-field images of cells treated with 10 μM RA along with either DMSO or 10 μM U0126 for 4 days. Means and SEM (error bars) were calculated from two independent experiments, each of which was conducted in triplicates. Quantification of the levels of NeuroD1 in *shScr* and *shWillin* cells from** (H)** Western blot analysis and **(I)** qPCR analysis. Means in **(H)** and **(I)** were calculated from two independent experiments conducted in triplicate. Error bars represent SEM. **(J)** Immunofluorescent imaging of *shScr* (upper row) and *shWillin* (lower row) cells for NeuroD1 (left column) and DNA (right column). Scale bars: 40 μm. **(K)** Quantification of extension bearing cells from bright-field images for *shScr* cells treated with 10 μM RA for 1, 4, and 7 days. Means and SEM (error bars) were calculated from two independent experiments, each of which was conducted in triplicates.** (L)** Development of the volume by which cells indent into the ERISM substrate over 1 week during RA-initiated (10 μM) neuronal differentiation of *shScr* cells. Data points represent means measured for *n*_i_ individual cells; shaded areas represent SEM (Day 1: *n*_shScr_ = 16, *n*_shScrRA_ = 31, *n*_shWillin_ = 22; Day 3: *n*_shScr_ = 21, *n*_shScrRA_ = 17, *n*_shWillin_ = 14; Day 6: *n*_shScr_ = 12, *n*_shScrRA_ = 16, *n*_shWillin_ = 19; Day 8: *n*_shScr_ = 18, *n*_shScrRA_ = 12, *n*_shWillin_ = 11). Groups were compared using Student’s *t*-test; ns: *p* > 0.05; *p ≤ 0.05; **p ≤ 0.01; ***p ≤ 0.001.

### Knock-Down of Willin/FRMD6 Alters Vinculin Expression, Focal Adhesion Assembly, and Actin Cytoskeleton Dynamics

To further investigate the effect of Willin/FRMD6 knockdown on exertion and transmission of cell force, *shScr* and *shWillin* cells were fixed on ERISM substrates and immunostained for the focal adhesion protein vinculin, the cytoskeleton protein actin and the mechanosensitive transcription co-activator TAZ (Figure 2A).

In line with the observation from the Fourier-filtered ERISM maps in Figure 1E, a decrease in Willin/FRMD6 expression resulted in the formation of fewer (mean number of focal adhesions per cell ± SEM: *shScr*: 30 ± 5, *n* = 24; *shWillin*: 16 ± 2, *n* = 37; Student’s *t*-test: *p* ≤ 0.01; Figures 2A,B) and smaller [mean average area of focal adhesion in cell ± SEM: *shScr*: (2.0 ± 0.2) μm^2^, *n* = 24; *shWillin*: (1.3 ± 0.1) μm^2^, *n* = 37; Student’s *t*-test: *p* ≤ 0.001; Figures 2A,C] focal adhesions. The position of vinculin in the fluorescence images co-localized with twisting in the Fourier-filtered ERISM maps, proving that the cells exert contractile forces *via* focal adhesion complexes. Western blotting showed a reduction in the overall vinculin expression (mean relative vinculin expression ± SEM: *shScr*: 1.00 ± 0.02, *n* = 3; *shWillin*: 0.63 ± 0.02, *n* = 3; Student’s *t*-test: *p* ≤ 0.001; Figure 2D). Also, *shWillin* cells showed the impaired formation of actin stress fibers (Figure 2A) and a reduction of the nuclear/cytosolic TAZ ratio (mean nuclear/cytosolic TAZ ratio ± SEM: *shScr*: 1.7 ± 0.1, *n* = 20; *shWillin*: 1.3 ± 0.1, *n* = 26; Student’s *t*-test: *p* ≤ 0.01; Figure 2E) while the total TAZ expression was unchanged (Supplementary Figure S3). Nuclear/cytosolic TAZ ratio roughly correlated with the number of focal adhesions and the mechanical activity for both *shScr* and *shWillin* cells (Figures 2F,G). While YAP RNA expression is generally low in SH-SY5Y cells, the amount of total YAP in Willin/FRMD6 knockdown cells was significantly diminished when compared to control cells (Supplementary Figure S3).

These data show that the knockdown of Willin/FRMD6 leads to general suppression of the mechanical activity of SH-SY5Y cells on various critical levels, as well as to morphological changes towards smaller but more elongated cells that form an increased number of small cell extensions.

### Willin/FRMD6 Overexpression Results in a General Reversion of the Findings Observed for Knock-Down Cells and Activate the Mechanical Activity of SH-SY5Y Cells

To investigate the effects of increased Willin/FRMD6 concentration, SH-SY5Y cells (*Willin*) overexpressing Willin/FRMD6 and reference cells (*Vector*) were generated by retroviral transfection. Western blot (mean relative Willin/FRMD6 expression ± SEM: *Vector*: 1.00 ± 0.06, *n* = 6; *Willin*: 2.40 ± 0.03, *n* = 6; Student’s *t*-test: *p* ≤ 0.001; Figure 3A) and qPCR (mean relative Willin/FRMD6 mRNA expression ± SEM: *Vector*: 1.00 ± 0.04, *n* = 6; *Willin*: 3.09 ± 0.07, *n* = 6; Student’s *t*-test: *p* ≤ 0.001; Figure 3B) were used to confirm successful Willin/FRMD6 overexpression.

An increase in Willin/FRMD6 led to a reversal of most of the findings made for Willin/FRMD6 knock-down cells. In detail, compared to *Vector* cells, *Willin* cells showed decreased proliferation (mean cell number after 8 days ± SEM: *Vector*: 25,100 ± 1,200, *n* = 9; *Willin*: 18,500 ± 2,400, *n* = 9; Student’s *t*-test: *p* ≤ 0.001; Figure 3C) and migration (mean number of migrating cells ± SEM: *Vector*: 313 ± 7, *n* = 6; *Willin*: 263 ± 6, *n* = 6; Student’s *t*-test: *p* ≤ 0.001; Figure 3D). However, no differences in vinculin expression and actin arrangement were found (Figure 3E). Furthermore, the observed increase in cell area [mean cell area ± SEM: *Vector*: (747 ± 62) μm^2^, *n* = 28; *Willin*: (802 ± 54) μm^2^, *n* = 37; Student’s *t*-test: *p* > 0.05; Figure 3F] and decrease in cell elongation (mean cell elongation factor ± SEM: *Vector*: 0.50 ± 0.05, *n* = 30; *Willin*: 0.43 ± 0.04, *n* = 37; Student’s *t*-test: *p* > 0.05; Figure 3G) of *Willin* cells compared to *Vector* cells were both not statistically significant (Supplementary Figure S2). Nuclear/cytosolic TAZ ratio (mean nuclear/cytosolic TAZ ratio ± SEM: *Vector*: 1.10 ± 0.02, *n* = 27; *Willin*: 1.24 ± 0.04, *n* = 29; Student’s *t*-test: *p* ≤ 0.001; Figure 3H) and cell force [mean indented volume ± SEM: *Vector*: (63 ± 8) μm^3^, *n* = 30; *Willin*: (91 ± 7) μm^3^, *n* = 31; Student’s *t*-test: *p* ≤ 0.01; Figure 3I], on the other hand, significantly increased in cells with higher Willin/FRMD6 expression.

### Knockdown of Willin/FRMD6 in SH-SY5Y Cells Activates the ERK1/2 Pathway and Elicits Differentiation Into a Neuronal Phenotype by Activation of the Transcriptional Factor NeuroD1

The changes in proliferation and morphology observed in the Willin/FRMD6 knockdown SH-SY5Y cells were reminiscent of ERK1/2-induced changes that have been observed in other neuronal cell lines (Gunn-Moore and Tavaré, 1998). We, therefore, explored whether basal ERK1/2 kinase activity was also changed in response to modulation of Willin/FRMD6 protein levels. Western blot analysis demonstrated that the knockdown of Willin/FRMD6 led to an increase of the amount of phosphorylated ERK1/2 compared to total ERK1/2 protein levels (mean relative phosphorylated ERK1/2 level ± SEM: *shScr*: 1.0 ± 0.1, *n* = 3; *shWillin*: 2.8 ± 0.3, *n* = 3; Student’s *t*-test: *p* ≤ 0.001; Figure 4A), while overexpression of Willin/FRMD6 had the opposite effect (mean relative phosphorylated ERK1/2 level ± SEM: *Vector*: 1.00 ± 0.02, *n* = 3; *Willin*: 0.66 ± 0.05, *n* = 3; Student’s *t*-test: *p* ≤ 0.001; Figure 4B). An increase in ERK1/2 phosphorylation was also observed in wild type SH-SY5Y cells that were treated with 10 μM RA, which is known to induce neuronal differentiation (mean relative phosphorylated ERK1/2 level ± SEM: DMSO: 1.00 ± 0.03, *n* = 3; RA: 2.46 ± 0.08, *n* = 3; Student’s *t*-test: *p* ≤ 0.001; Figure 4C).

We next examined whether the morphological and biochemical effects observed in the Willin/FRMD6 knockdown SH-SY5Y cells were dependent on active ERK signaling. For this, we used U0126, a highly selective MEK inhibitor that prevents the activation of ERK1/2 (Favata et al., 1998). Inhibition of MEK by U0126 treatment (10 μM) was indeed effective at blocking the activation of ERK1/2 caused by the decrease of Willin/FRMD6 expression in *shWillin* cells (mean relative phosphorylated ERK1/2 level ± SEM: DMSO: 1.00 ± 0.03, *n* = 3; U0126: 0.13 ± 0.01, *n* = 3; Student’s *t*-test: *p* ≤ 0.001; Figure 4D). Blocking ERK1/2 activation *via* U0126-mediated MEK inhibition reduced the formation of neurite-like extensions in Willin/FRMD6 knock-down cells down to the level of control cells (mean percentage of cells with neurite-like extensions ± SEM: *shScr-DMSO*: 1.3% ± 0.3%, *n* = 6; *shWillin-U0136*: 2.3% ± 0.8%, *n* = 6; *shWillin-DMSO*: 13.3% ± 0.8%, *n* = 6; Student’s *t*-test: *p(shScrDMOS-shWillinU0126)* > 0.05, *p(shWillinU0126-shWillinDMSO)* ≤ 0.001; Figure 4E and Supplementary Figure S4) and returned the cell force of Willin/FRMD6 knock-down cells back to the level of control cells [mean indented volume ± SEM: *shScr-DMSO*: (120 ± 12) μm^3^, *n* = 34; *shWillin-U0126*: (95 ± 8) μm^3^, *n* = 54; *shWillin-DMSO*: (75 ± 7) μm^3^, *n* = 44; Student’s *t*-test: *p(shScrDMSO-shWillinU0126)* > 0.05, *p(shWillinDMSO-shWillinU0126)* ≤ 0.05; Figure 4F]. Blocking ERK1/2 activation with U0126 also stalled RA-induced differentiation of control and Willin/FRMD6 knocked down cells, which indicates that U0126 treatment directly affects differentiation of SH-SY5Y cells (mean percentage of differentiated cells after 4 days ± SEM: *shScr-DMSO*: 8.6% ± 1.5%, *n* = 6; *shScr-U0136*: 5.1 ± 0.2%, *n* = 6; *shWillin-DMSO*: 19.1% ± 1.5%, *n* = 6; *shWillin-U0136*: 4.6% ± 0.5%, *n* = 6; Student’s *t*-test: *p(shScrDMSO-shScrU0126)* ≤ 0.05, *p(shWillinDMSO-shWillinU0126)* ≤ 0.001; Figure 4G). These findings suggest that the reduction of Willin/FRMD6 protein levels in SH-SY5Y cells leads to the activation of known signaling transduction pathways and promotes neuronal differentiation.

We looked at NeuroD1 levels to test this hypothesis. NeuroD1 is a transcriptional factor activated downstream of ERK1/2 (Petersen et al., 2002). It is known to be upregulated upon RA-induced differentiation of SH-SY5Y cells and is thus associated with control of neurite outgrowth (López-Carballo et al., 2002). NeuroD1 has also been reported to translocate to the nucleus after its phosphorylation by ERK2. For this reason, we explored whether the knockdown of Willin/FRMD6 and associated activation of ERK1/2 was able to phenocopy the RA-mediated increase of NeuroD1 expression. An elevation in NeuroD1 expression was observed in the Willin/FRMD6 knockdown cells using both Western blot (mean relative NeuroD1 expression ± SEM: *shScr*: 1.0 ± 0.1, *n* = 6; *shWillin*: 1.7 ± 0.2, *n* = 6; Student’s *t*-test: *p* ≤ 0.001; Figure 4H) and qPCR analysis (mean relative NeuroD1 mRNA expression ± SEM: *shScr*: 1.00 ± 0.02, *n* = 6; *shWillin*: 16.00 ± 0.02, *n* = 6; Student’s *t*-test: *p* ≤ 0.001; Figure 4I). Furthermore, knockdown of Willin/FRMD6 led to a change in cellular localization of NeuroD1, specifically an increased presence of NeuroD1 in the cell nucleus (Figure 4J).

Next, *shScr* and *shWillin* cells were exposed to RA over 7 days to induce neuronal differentiation (Supplementary Figure S5). Figure 4K shows that the proportion of cells with growing neurite-like extensions was approximately doubled in cells with low Willin/FRMD6 expression after 4 and 7 days (percentage of cells with neurite-like extensions after 7 days ± SEM: *shScr*: 30% ± 1%, *n* = 6; *shWillin*: 49% ± 4%, *n* = 6; Student’s *t*-test: *p* ≤ 0.001), which is in-line with the hypothesis that increased activation of ERK1/2 in these cells make them more susceptible for RA-induced neuronal differentiation (Figure 4A). Inhibition of ERK1/2 blocked the RA-induced neurite elongation in both cell types (Figure 4G), which suggested that activation of ERK1/2 is the main mechanism regulating neuronal differentiation. The enhanced neurite growth was specific to a reduction of Willin/FRMD6 expression; increasing Willin/FRMD6 expression did not affect RA induced differentiation of SH-SY5Y cells (data not shown). Also, when followed over 1 week, the mechanical activity of Willin/FRMD6 knockdown cells converged towards the mechanical activity of control cells for which neuronal differentiation was induced by RA treatment [mean indented volume after 8 days ± SEM: *shScr*: (74 ± 11) μm^3^, *n* = 18; *shScr-RA*: (16 ± 4) μm^3^, *n* = 12; *shWillin*: (29 ± 5) μm^3^, *n* = 11; Student’s *t*-test: *p(shScr-shScrRA)* ≤ 0.001, p(shScrRA-shWillin) > 0.05; Figure 4L]. This shows that knockdown of Willin/FRMD6 phenocopies the mechanical activity of RA-induced neuronal differentiation.

## Discussion

The FERM domain-containing protein Willin/FRMD6 has previously been shown to be an upstream activator of the Hippo signaling pathway in a variety of different cell types (Moleirinho et al., 2013a; Gunn-Moore et al., 2016). Even though Willin/FRMD6 was originally discovered in the rat sciatic nerve and subsequently has been shown to affect peripheral neuronal formation and function (Moleirinho et al., 2013b; Fernando et al., 2016), most studies have focused on its functional roles in cells outside of the nervous system.

Here we showed that the neuroblastoma cell line SH-SY5Y, which is a widely used neuronal model cell system, expresses the Willin/FRMD6 protein at a level comparable to primary mouse cortical neurons. Our data suggest that the Willin/FRMD6 expression level influences cellular, biophysical, and biochemical changes in SH-SY5Y cells. Specifically, reducing the endogenous levels of Willin/FRMD6 led to an increase in the proliferation and migration capacity and to pronounced changes in the morphology of SH-SY5Y cells, such as cell elongation and the production of fine extensions. Links between Willin/FRMD6 and cell proliferation (Moleirinho et al., 2013c), cell migration (Moleirinho et al., 2013c; Wang et al., 2019) or cell morphology have been noted for other cell types before Angus et al. (2012), Moleirinho et al. (2013c) and Gunn-Moore et al. (2016). In all the reported cases, the observed changes were attributed to the function of Willin/FRMD6 as an upstream activator of the Hippo signaling pathway.

On the cell mechanical level, knocking down Willin/FRMD6 expression in SH-SY5Y cells resulted in the impaired organization of actin stress fibers; a reduction in expression levels of vinculin and thus the formation of fewer and smaller focal adhesions; a change in the localization of TAZ; and reduction of the force the cells exerted on their substrate. Mechanistically, it is conceivable that either the exclusion of the transcription cofactor TAZ from the nucleus could result in the transcriptional regulation of the cytoskeleton and focal adhesion, or that reduced formation of focal adhesions regulates the cytoskeleton which in turn regulates TAZ localization (Totaro et al., 2018). The mechanism by which Willin/FRMD6 impacts the mechanical phenotype of SH-SY5Y cells still needs to be further examined. The FERM protein domain is generally involved in localizing proteins, especially cytoskeletal-associated proteins, to the plasma membrane (Chishti et al., 1998). It was previously shown that Willin/FRMD6 co-localizes with actin at the plasma membrane (Gunn-Moore et al., 2005), and Willin/FRMD6 was previously reported to modulate cellular F-actin organization in other cell types (Gunn-Moore et al., 2016). In epithelial cells, Willin/FRMD6 localizes at apical junctional complexes, where it regulates contractility of the circumferential actomyosin cables *via* recruitment of aPKC that in turn mediates phosphorylation of ROCK (Ishiuchi and Takeichi, 2011). Willin/FRMD6 may have a similar role in focal adhesion complexes of SH-SY5Y cells.

Our data show at the biochemical level, that the reduction of endogenous Willin/FRMD6 protein level leads to the activation of the ERK1/2 signaling transduction pathway with a correlative increase in expression and change in cellular location of the transcriptional factor NeuroD1. This seems to prime SH-SY5Y cells for RA-induced neuronal differentiation. When kept in culture over an extended period, SH-SY5Y cells with reduced Willin/FRMD6 expression phenocopied RA-induced neuronal differentiation and showed similar levels of cell forces. The observed changes in morphology and force exertion of Willin/FRMD6 knockdown cells were suppressed in the presence of the specific ERK1/2 inhibitor U0126, which suggests that the cross-talk occurs upstream of the kinase, probably at the Ras/Raf level as previously proposed (Romano et al., 2014). Our findings are consistent with earlier reports showing that components of the Hippo signaling pathway are linked to the ERK pathway (Zhang et al., 2009; Reddy and Irvine, 2013; Romano et al., 2014). Activation of ERK signaling and consequential enhancement of induced neuronal differentiation has also been reported upon the downregulation of the ERK-inhibitor Spry4 in PC12 cells (Alsina et al., 2012) and upon increased interaction with the neural cell adhesion molecule NCAM in SH-SY5Y cells (Seidenfaden et al., 2006). Contrary to our observation that activation of ERK in Willin/FRMD6 knockdown cells resulted in reduced cell force exertion, ERK has been reported to trigger cell contractility in a non-neuronal cell type (Hino et al., 2020).

We also found that an increase of Willin/FRMD6 concentration, on the other hand, triggered the opposite cellular, morphological, mechanical, and biochemical changes. However, the changes in phenotype observed for knock-down cells were more pronounced than the reversed effect seen in overexpressing cells.

While all observations suggest that Willin/FMRD6 knockdown promotes differentiation of SH-SY5Y cells, the functional relationship between the observed phenomena and the potential involvement of the Hippo pathway will still have to be investigated in the future. We show that Willin/FRMD6 affects the nucleocytoplasmic shuttling of TAZ in SH-SY5Y cells, even though the nuclear/cytoplasmic TAZ ratio in Willin/FMRD6 knockdown cells was only reduced by 25% compared to control cells. Neuronal differentiation of SH-SY5Y cells in conjunction with cytoplasmic retention of TAZ as a result of activation of Hippo signaling by the cadherin FAT1 has been reported previously (Ahmed et al., 2015). It is conceivable that Willin/FMRD6, which has been reported to play a role in cell-cell contacts (Ishiuchi and Takeichi, 2011), has a similar function. While total TAZ expression in SH-SY5Y cells was not affected by Willin/FRMD6 knockdown, the observed reduction of the already low total YAP expression in Willin/FRMD6 knockout cells might support the assumption that Hippo signaling could be interrupted by Willin/FRMD6 knockdown and that alternative pathways might be involved. Downregulation of YAP protein expression upon upregulation of Neurog2 (a member of the same subgroup of bHLH transcription factors for neurogenesis like NeuroD1) has been reported in mouse retinal progenitor cells, and it was proposed that mutual inhibition between proneural bHLH proteins and YAP is an important regulator of proliferation and cell cycle exit during mammalian neurogenesis (Zhang et al., 2012). By contrast, our data show that Willin/FRMD6 knockdown not only promotes neuronal differentiation of SH-SY5Y cells but also results in increased proliferation.

Future work will also have to test whether the link between Willin/FRMD6 and ERK1/2-activated differentiation also occurs in primary neuronal cells. However, the circumstantial evidence that Willin/FRMD6—like other upstream components of the Hippo pathway—may be linked to neurodegenerative diseases, leads to the intriguing hypothesis that Willin/FRMD6 may play a role in the pathogenesis of these diseases. Indeed, Willin/FRMD6 expression has been reported to be down-regulated in Alzheimer’s disease models (Castillo et al., 2017). Our work, therefore, opens the door for future studies on how Willin/FRMD6 may not only influence neuronal differentiation but may also play a role in neurodegeneration.

## Data Availability Statement

All data needed to evaluate the conclusions in this article are presented in the article and/or Supplementary Materials. The research data supporting this publication can be accessed at: https://doi.org/10.17630/e0983924-b0fc-41ac-9c05-35758150df00.

## Author Contributions

All authors contributed to the project and experimental design. NK, AT-L, FT, DC and WY conducted experiments and analyzed data. KD, MG and FG-M supervised experiments. NK and AT-L wrote the first draft of the manuscript with contributions by MG and FG-M. All authors contributed to the article and approved the submitted version.

## Conflict of Interest

The authors declare that the research was conducted in the absence of any commercial or financial relationships that could be construed as a potential conflict of interest.
