# Supplementary material for: Willin/FRMD6 Influences Mechanical Phenotype and Neuronal Differentiation in Mammalian Cells by Regulating ERK1/2 Activity
Source: Front Cell Neurosci. 2020 Sep 4;14:552213. doi: 10.3389/fncel.2020.552213 (PMC7498650; doi:10.3389/fncel.2020.552213)
Supplement: Supplementary file 1 [file Presentation_1.pdf]

Supplementary Materials

Willin/FRMD6 influences mechanical phenotype and neuronal differentiation in mammalian cells by regulating ERK1/2 activity

Nils M. Kronenberg*[,1,2], Andrew Tilston-Lunel*[,3,4], Frances E. Thompson[1], Doris Chen[3], Wanjia Yu[3], Kishan Dholakia[1,5], Malte C. Gather[1,2,#] and Frank J. Gunn-Moore[3,#]

1) Centre of Biophotonics and SUPA, School of Physics and Astronomy, University of St Andrews, St Andrews, UK
2) Centre for Nanobiophotonics, Department of Chemistry, University of Cologne, Cologne, Germany
3) Centre of Biophotonics, School of Biology, University of St Andrews, St Andrews, UK
4) Department of Biochemistry, School of Medicine, Boston University, Boston, USA
5) Department of Physics, College of Science, Yonsei University, Seoul, South Korea

* These authors have contributed equally to this paper
\# correspondence to: mcg6@st-andrews.ac.uk, fjg1@st-andrews.ac.uk

Content:

- **Supplementary Figure 1 |** SH-SY5Y cells and primary mouse cortical neurons exhibit identical Willin/FRMD6 expression levels.
- **Supplementary Figure 2 |** Willin regulates the morphology of SH-SY5Y cells.
- **Supplementary Figure 3 |** Knockdown of Willin/FRMD6 does not affect total TAZ but reduces total YAP expression in SH-SY5Y cells.
- **Supplementary Figure 4 |** Blocking ERK1/2 activation via U0126-mediated MEK inhibition reduced the formation of neurite-like extensions in Willin/FRMD6 knock-down SH-SY5Y cells.
- **Supplementary Figure 5 |** RA-induced differentiation of SH-SY5Y cells.
- **Supplementary Table 1 |** Primers used for qPCR analysis.

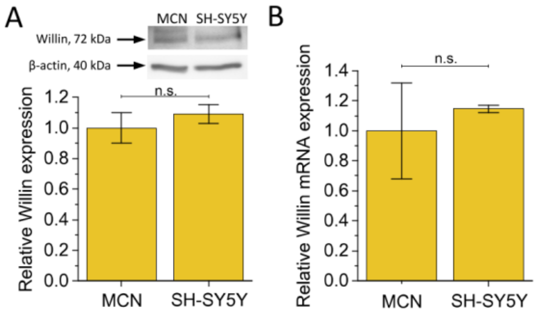

Supplementary Figure 1 | SH-SY5Y cells and primary mouse cortical neurons exhibit identical Willin/FRMD6 expression levels. **(A)** Quantitative Western blot analysis of Willin/FRMD6 expression in primary mouse cortical neurons (MCN) and SH-SY5Y cells. Means and SEM (error bars) were calculated from three independent experiments. **(B)** qPCR analysis of Willin/FRMD6 mRNA expression in primary mouse cortical neurons (MCN) and SH-SY5Y cells. Means and SEM (error bars) were calculated from two independent experiments, each of which was conducted in triplicates. Groups were compared using Student's *t*-test; n.s.: $p > 0.05$.

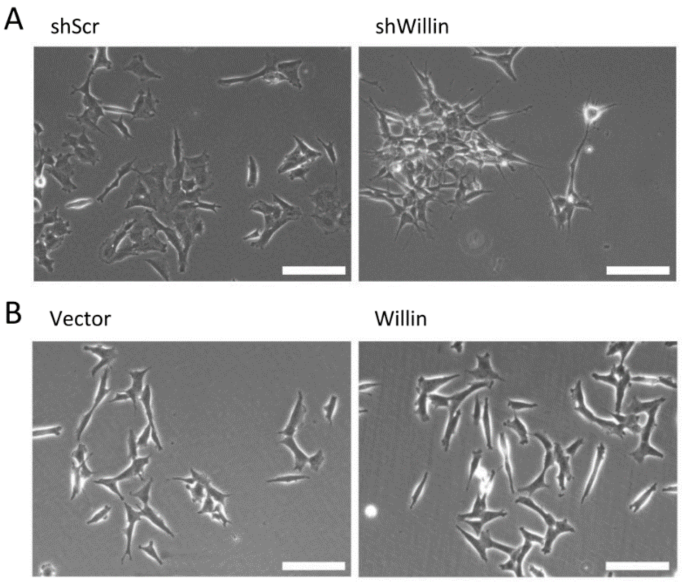

Supplementary Figure 2 | Willin regulates the morphology of SH-SY5Y cells. (A) Representative bright field images of *shScr* and *shWillin* cells grown in standard plastic cell culture dishes demonstrating the effect of Willin depletion on cellular morphology. Neurite-like extensions are observed for *shWillin* cells. (B) Representative bright field images of *Vector* and *Willin* cells grown in standard plastic cell culture dishes demonstrating that the overexpression of Willin has no effect on the morphology of SH-SY5Y cells. Scale bars: 20 µm.

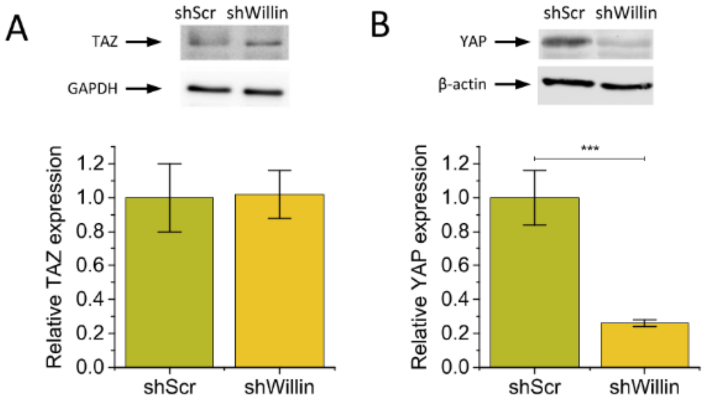

Supplementary Figure 3 | Knockdown of Willin/FRMD6 does not affect total TAZ but reduces total YAP expression in SH-SY5Y cells. Quantitative Western blot analysis of **(A)** TAZ and **(B)** YAP expression in *shScr* and *shWillin* cells. Means were calculated from three technical repeats. Error bars represent ±SEM. Groups were compared using Student's *t*-test; ***: $p \leq 0.001$.

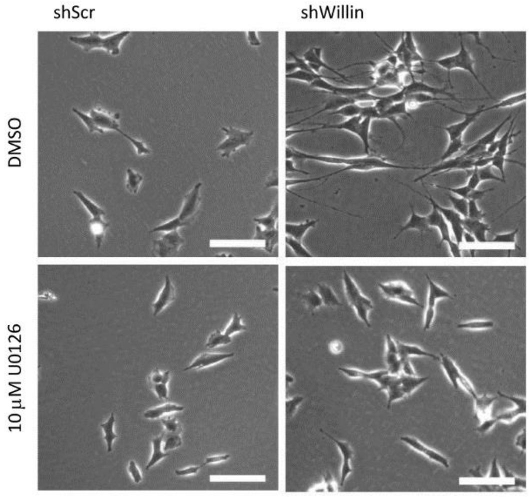

Supplementary Figure 4 | Blocking ERK1/2 activation via U0126-mediated MEK inhibition reduced the formation of neurite-like extensions in Willin/FRMD6 knock-down SH-SY5Y cells. Representative bright field images of *shScr* and *shWillin* cells grown in standard plastic cell culture dishes that were treated with DMSO or 10 μM U0126 for 24 hours. Scale bars: 20 μm.

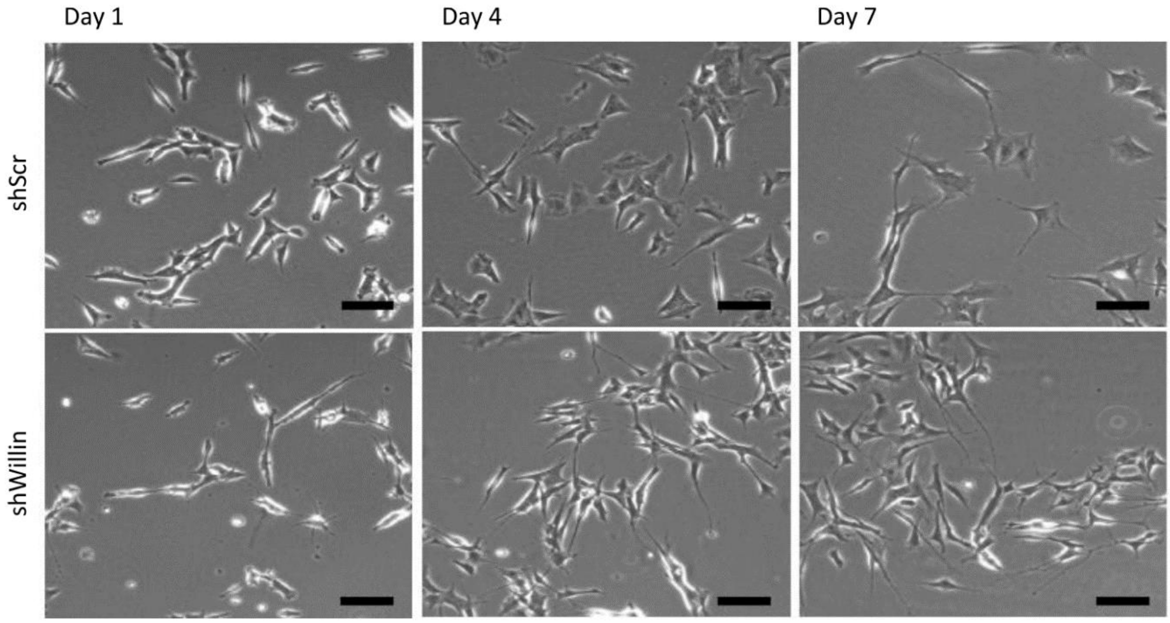

Supplementary Figure 5 | RA-induced differentiation of SH-SY5Y cells. Representative bright field images of *shScr* and *shWillin* cells grown in standard plastic cell culture dishes before treatment with RA and after 4 and 7 days of treatment with 10 µM RA. RA treatment of *shWillin* cells increases the number of cells undergoing differentiation compared to *shScr* cells. Differentiated cells were defined as cells with neurites that were longer than 40 µm (see Methods and Materials). Scale bars: 20 µm.

Supplementary Table 1 | Primers used for qPCR analysis.

Oligo	Sequence (5'-3')
hWillin FW	TGAAAACCTGCAGCTCAATG
hWillin RV	CTCTGGCCACGAAGCTTAAC
mWillin Fw	CGGCAATACGAAGTCACTTGGG
mWillin RV	TGCAATTCGGTCACTGATCAGC
HNeuroD1 FW p1	GCGGCCCCAAAAAGAAGAAG
HNeuroD1 FW p2	AGCCCTCTGACTGATTGCAC
HNeuroD1 RV p1	TCCGACAGAGCCCAGATGTA
HNeuroD1 RV p2	GTCTATGGGGATCTCGCAGC
